# Thirteen kinds of Chinese medicine injections for acute exacerbation of chronic obstructive pulmonary disease: Protocol for a systematic review and network meta-analysis

**DOI:** 10.1097/MD.0000000000016200

**Published:** 2019-06-28

**Authors:** Wenjiang Zheng, Tianqi Gao, Huiting Huang, Zhihui Zhou, Qian Yan, Yu Hong, Huili Liao, Tiange Zhang, Xiaohong Liu

**Affiliations:** a Guangzhou University of Chinese Medicine; b The First Affiliated Hospital of Guangzhou University of Chinese Medicine, Guangzhou; c The Dongguan hospital of Chinese Medicine, Dongguan, China.

**Keywords:** acute exacerbation of chronic obstructive pulmonary disease, Chinese medicine injections, network meta-analysis, protocol, systematic review

## Abstract

**Background:**

Chinese medicine injections (CMIs) are extensively applied to the therapy of acute exacerbation of chronic obstructive pulmonary disease (AECOPD) in mainland China. Up to 13 different kinds of CMIs are reportedly often used for treating chronic obstructive pulmonary disease, yet, rarely head to head comparison of tests are used to decide the relative consequent among the distinct CMIs. Network meta-analysis (NMA) will be performed to further compare the effects of 13 different CMI, including direct and indirect comparisons of different CMI.

**Methods:**

From now until April 2019, a systematic and comprehensive literature search will be conducted in both English and Chinese databases, including Medline, Embase, Cochrane library, Chongqing VIP information, Wanfang Database, China national knowledge infrastructure database, and Sino Med. Randomized controlled trials will be included related to CMI therapy for AECOPD. We will assess the quality of the included trials in accordance with the risk of bias tools in Cochrane manual 5.1.0. We will use the grading of recommendations assessment development, and evaluation method to assess the certainty of the estimated evidence from the NMA. STATA 14.0 will be used for data analysis.

**Results:**

The purpose of this systematic evaluation and NMA was to summarize and rank the direct and indirect evidence for 8 different types of CMI. The NMA's findings will be reported in accordance with preferred reporting items for systematic reviews and meta analyses-NMA statement. Upon completion, NMA results will be submitted to a peer-reviewed journal.

**Conclusion:**

With NMA, this study will provide evidence for the selection of CMI for patients with AECOPD. The results will provide information to clinicians, bridge the evidence gap and identify promising CMI targets for future trials.

**PROSPERO registration number:**

PROSPERO CRD 42019132955.

## 1 Introduction

### 1.1 Basic characteristics of chronic obstructive pulmonary disease and the conventional treatment

Chronic obstructive pulmonary disease (COPD) is a disease characterized by persistent respiratory symptoms and restricted airflow,^[1]^ and is one of the leading causes of increased morbidity and mortality worldwide. This situation is getting worse, the number of patients with COPD in China has reached 100 million,^[2]^ causing serious economic and social burdens. Acute exacerbation of chronic obstructive pulmonary disease (AECOPD) is a serious event in the management of COPD.^[3]^ At this time, the patient's respiratory symptoms deteriorate acutely, leading to the need for additional treatment. The most common cause is a respiratory infection, and repeated acute attacks will accelerate disease progression. At the same time, AECOPD's current western medicine treatment programs include anti-infective, anti-inflammatory, and anti-inflammatory asthma, dilated bronchial and mechanical ventilation therapy. Glucocorticoid is currently the most potent anti-inflammatory drug, and the correct use of glucocorticoids can benefit patients a lot. However, many studies have shown that hormones cannot effectively control the progressive development of airway inflammation in the treatment of COPD, nor can it reverse the decline of lung function, and the phenomenon of hormone therapy is not sensitive.^[4]^

The AECOPD is a continuation of the hormone-insensitive state in the stable phase. At the same time, long-term repeated respiratory infections in patients with COPD can cause immune dysfunction in the body. Studies have shown that immune imbalance is considered to be an important pathophysiologic basis for the continuous progression of COPD.^[5]^ Cellular and humoral immunity of patients with COPD are damaged to varying degrees, resulting in pathogens. Microorganisms can easily invade and induce infection, and prolonged and difficult to heal, suggesting that the occurrence and outcome of the disease is closely related to the body's immune function. Therefore, the efficacy of conventional western medicine treatment needs to be improved.

### 1.2 Application of Chinese medicine injections for AECOPD

A number of systematic reviews^[6–9]^ have suggested that the combination of traditional Chinese medicine injections (CMI) on the basis of conventional western medicine treatment can not only improve the clinical efficacy of the disease, but also enhance the immune function of patients, and have the synergistic effect of antiinflammatory and antiasthmatic effects in recent years. Because of the clinical use of various CMI, clinicians are challenged to select the best CMI for patients with AECOPD. A number of randomized controlled trials (RCTs) and systematic evaluations have assessed the efficacy of various CMI for AECOPD. Nevertheless, most of these studies were designed to compare with traditional western medicine. Few studies compare different CMI. Thence, the efficiency of comparison between different CMI is still uncertain. Therefore, we intend to conduct a systematic evaluation and a network meta-analysis (NMA) to compare the efficacy of 13 different CMI and rank the benefits among them. We hope that the results of this study will contribute to the management and application of CMI in the treatment of AECOPD.

## 2 Methods

### 2.1 Study registering and reporting

The research will follow the preferred reporting items for systematic reviews and meta analyses (PRISMA) for NMA guidelines for reporting the results of the review. PROSPERO (international register of expectations system evaluation) (CRD42019132955) has registered this plan. We will record any protocol changes made during the implementation of the review in the publication of the final report. The PRISMA extension declaration is a declaration that ensures that all aspects of the method and result are reported.^[10]^ We followed the PRISMA-P guidelines.^[11]^

### 2.2 Eligibility criteria

The eligibility criteria for the review using the PICOS (population-intervention-comparative-results-study design) framework are as follows.

### 2.3 Population

We will consider RCTs with a clear diagnosis of AECOPD. Most of the studies published in China will use the revised criteria from the consensus of Chinese experts on acute exacerbation of COPD in 2017.^[3]^ There will be no limits on age, gender, race or nationality.

### 2.4 Interventions/comparators

In our preliminary analysis of the literature related to the treatment of acute cerebral infarction, we found that 13 CMIs were most commonly used to treat AECOPD with antibacterial, antiviral, antiinflammatory, and asthma functions. All 13 types of CMI can improve clinical symptoms. Table 1 lists the basic information for the 13 CMI types. The routine treatment has been defined as oxygen absorption, spasm lysis, antiasthma, antiinfection, antiinflammatory, and nutritional support for the convenience of data analysis. The comparison of suitable conditions is as follows: CMIa + conventional treatment and CMIb + conventional treatment; CMI + compared with conventional treatment. In view of that that western medicines have been rapidly updated and some of them have been withdrawn from the market, studies on CMI with very specific western medicines will be excluded. There is no limit to the dosage or course of treatment.

**Table 1:**
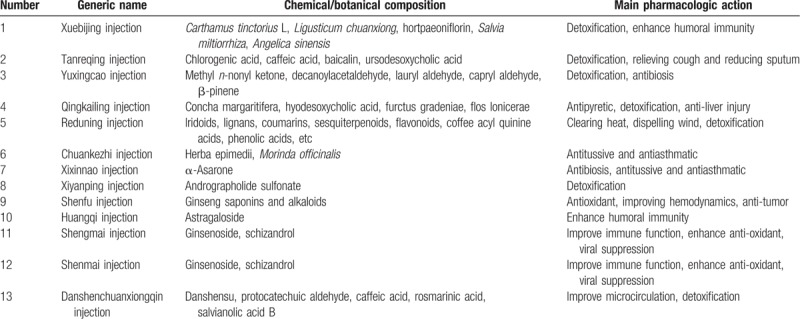
Basic information on the 13 kinds of Chinese medicine injections to be included.

### 2.5 Outcome measures

Clinical trials of AECOPD published in academic journals were reviewed and found to have regularly reported results: Pulmonary function is the gold standard for the diagnosis of COPD, and it is also an important part of disease prognosis and treatment evaluation. The percentage of forced expiratory volume in 1 second (FEV_1_) in lung function is still the most important lung function index in current clinical examination, and it is the diagnosis of chronic obstructive pulmonary disease and airflow limitation.^[12]^ The gold standard for severity assessment is also one of the most important indicators for clinical efficacy assessment. Therefore, this study used FEV_1_ as the main efficacy index.

### 2.6 Study design

Strict inclusion/exclusion criteria have been defined for this study to limit heterogeneity and enhance clinical applicability. Only RCTs associated with CMI in AECOPD therapy were included for analysis. We will exclude duplicate studies that do not have sufficient information to calculate the effect estimates. We will not apply any language or other restrictions to this.

### 2.7 Search strategy

We will start from the database by searching four Chinese databases (China national knowledge infrastructure database, wan fang database, Chongqing VIP information, and SinoMed) and three English databases (Medline, Cochrane library, and Embase) to ensure that all possible AECOPD and CMI studies are included. Targeted grey literature searches will be conducted against the Clinical trials.gov and the international Clinical trial registration platform retrieval portal to identify ongoing and completed trials. In these databases, restrictions will be placed to exclude case reports, meeting abstracts, reviews, news articles, bibliographies, book chapters, biographies, letters, reference material, and editorials. Key search terms will include all drug names developed within the 2 drug classes being assessed. These are based on previous systematic reviews conducted in this area.^[6–9]^ In all database searches, there will be no restrictions on languages. However, once the search has been conducted, papers that are not in English or Chinese will be excluded. The search strategy of Medline is as follows:

- #1 Search (“Pulmonary Disease, Chronic Obstructive”[MeSH]) OR ((“COPD” or “Chronic Obstructive Pulmonary Disease” or “COAD” or “Chronic Obstructive Airway Disease” or “Chronic Obstructive Lung Disease” or “Airflow Obstruction, Chronic” or “Airflow Obstructions, Chronic” or “Chronic Airflow Obstructions” or “Chronic Airflow Obstruction”))
- #2 Search (“Medicine, Chinese Traditional” [MeSH]) OR ((“Traditional Chinese Medicine” or “Chung I Hsueh” or “Hsueh, ChungI” or “Traditional Medicine, Chinese” or “Zhong Yi Xue” or “Chinese Traditional Medicine” or “Chinese Medicine, Traditional” or “ Chinese patent medicine” or “Chinese patent drug” or “proprietary Chinese medicine” or “proprietary Chinese drug” or “Chinese heral injection” or “Chinese medicine injection”))
- #3 Search (“Injections”[MeSH]) OR ((“Injection” or “Injectables” or “Injectable”))
- #4 #2 AND #3
- #5 Search ((“Xuebijing injection” or “Tanreqing injection” or “Yuxingcao injection” or “Qingkailing injection” or “Reduning injection” or “Chuankezhi injection” or “Xixinnao injection” or “Xiyanping injection” or “Shenfu injection” or “Huangqi injection” or “Shengmai injection” or “Shenmai injection” or “Danshenchuanxiongqin injection”))
- #6 #4 OR #5
- #7 #1 AND #6

### 2.8 Study selection and data extraction

**Figure 1:**
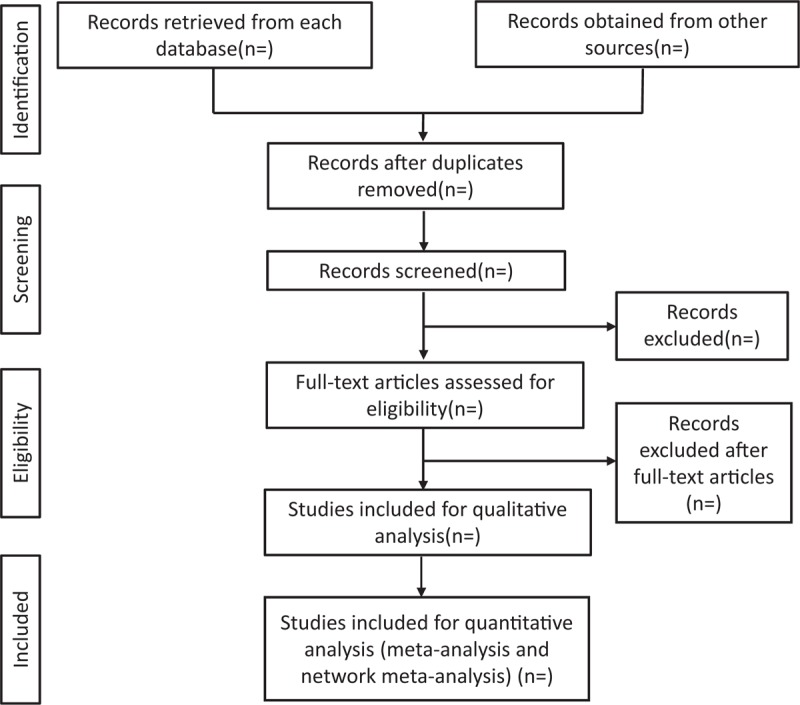
Preferred reporting items for systematic review and meta-analysis flow chart of study selection process.

Once the search has been completed, papers will be imported into EndNote X9.0. For each study, the title, authors, year of publication, the language of paper, type of publication, journal, volume, issue, and pages will be extracted, where duplicate papers across the three database searches will be removed. Once duplicates have been removed, 2 independent reviewers will screen titles and abstracts, excluding nonrelevant papers according to the inclusion/exclusion criteria. If it is unclear whether a paper has met the inclusion/exclusion criteria, this will be selected for full-text screening. Disagreements on papers for full-text screening between the 2 reviewers will be resolved by a 3rd reviewer. Data will then be extracted from relevant papers selected after full-text screening. Additionally, references of included papers will be searched for any other appropriate papers that may have been missed previously. Risk of bias for each trial included will also be assessed. To ensure the quality of extraction, a random sample of 10% of the included trials will be selected and another reviewer will independently extract data and assess the risk of bias. The agreement between the primary researcher and the 2nd reviewer will be compared; if the level of agreement is below 80%, a full independent duplicate extraction will be conducted. The screening process will be presented with reference to the PRISMA statement as Figure 1.

We will use Microsoft Excel 2013 to extract data and collect relevant information. The extracted information will be classified into 5 parts: publication information: publication year, 1st author, publication, and journal country; details of intervention and control therapy: dosages, drugs, treatment course, follow-up; general characteristics of patients: disease, age, gender, sample size, eligibility criteria, numbers of dropouts, and baseline information; details of outcomes: respiratory function evaluation index (the percentage of FEV_1_) and the concentrated evaluation index of expert advices included arterial oxygen pressure, asthmatic, artery CO_2_ pressure, etc.^[13]^

### 2.9 Quality assessment

The risk of bias will be assessed using the Risk of Bias Tool (ROB) in Cochrane Handbook 5.1.0.^[14]^ The sources of bias assessed will include whether a random sequence generation was used, if treatment allocation was concealed, blinding of participants, and researchers from what treatment participants received, the incompleteness of any primary outcomes and selectivity of reporting. Each of the sources of bias in each trial will be assessed and classed as either “high risk,” “low risk,” or “unclear risk,” where the percentages for each category in each source of bias analyzed will be described and the results interpreted taking the risk of bias into account. Sensitivity analysis will also be conducted excluding studies reporting the high risk of bias in any domain analyzed. We will assess the certainty of evidence contributing to network estimates of the primary outcome through the grading of recommendations assessment, development, and evaluation (GRADE) system.^[15]^ Based on 5 key areas (bias, indirection, inconsistency, imprecision, and risk of publication bias), the quality of evidence is divided into one of 4 levels: high, medium, low, and very low.

### 2.10 Statistical analysis

#### 2.10.1 Pairwise meta-analysis

The conventional pairwise meta-analysis will be completed by Stata 14.0 software. The pooled odds ratios with 95% confidence interval (CI) are calculated for dichotomous data weighted mean difference with 95% CI are calculated for continuous data (FEV_1_). For detecting the potential heterogeneity across the included studies, we will conduct the Chi-squared test and *I*^2^ test. If *I*^2^ < 50% and *P* > .05, it shows that heterogeneity does not matter and that the mantel-haenszel fixed model will be used for the meta-analysis. If *I*^2^ ≥ 50% and *P* ≤ .05, it indicates the need to analyze heterogeneity. There are 3 kinds of heterogeneity, statistical heterogeneity, clinical heterogeneity, and methodologic heterogeneity.^[16]^ For statistical heterogeneity, the random effects model will be used. If clinical and methodologic heterogeneity exists, subgroup analysis or meta-regression analysis will be performed. If the source of heterogeneity is unknown, we will abandon the synthetic analysis and adopt descriptive analysis. Sensitivity analysis will be used to incorporate robustness of the results. Sensitivity analysis will be employed for the robustness of results of the included studies. If the number of studies is no <10, the funnel plot will be used to detect publication bias in the trials included in the current NMA. We will perform Egger test for detecting asymmetry through a funnel plot.^[17]^

#### 2.10.2 Network meta-analysis

The NMA will be conducted using the network command in STATA 14.0.^[18–21]^ STATA will also be used in the drawing of Network Plots of Network Meta. Then we will conduct a heterogeneity test and an inconsistency test. If there is a closed loop, we will use the inconsistency factor to assess the heterogeneity between the included studies. If the 95% CIs of the inconsistency factor is truncated at 0, it indicates that the direct evidence and the indirect evidence are consistent.^[22]^ To rank probabilities of treatments, the surface under the cumulative ranking (SUCRA) will be used to summarize the probability values. SUCRA value is 100% for optimal treatment and 0% for worst treatment.^[23]^ Funnel plots will be compared and adjusted to assess the presence of small-study effects.^[23]^In addition, due to the quality level of evidence at each outcome often differs, we will produce and perform a summary of the evidence with the Grading of Recommendations, Assessment, GRADE. All data will be processed through STATA software (version 14.0).

### 2.11 Patient and public involvement

This part is not covered in this study.

## 3 Discussion

### 3.1 Advantage of the NMA

Chinese herbal medicine injection is derived from the effective substance extracted and purified from herbal medicine (or decoction tablets). CMIs can be made from plants or herbs to animals, so they have a wider range of sources. During the process of retrieving literature, only one NMA about Chinese herbal injections (CHIs) for AECOPD was detected.^[24]^ We compared the current NMA with the other NMA to demonstrate these differences. From the details of the comparison that been shown in Table 2, the current NMA will update the literature retrieve and focus on 2 different interventions for the treatment of AECOPD. By GRADE assessment, evidence certainty will be incorporated into the main results of the current NMA to highlight the most robust findings for further use. Although systematic evaluation and randomized controlled trials of CMI therapy for AECOPD are available, it is challenging for clinicians to select the best CMI for AECOPD management because the conventional head-to-head comparisons between CMIs for AECOPD are not sufficient. Emerging NMA can be used to compare multiple interventions simultaneously and analyze studies by comparing them differently in the same analysis. Just like the NMA above, NMA has already been used in evaluating CHIs for COPD.^[24]^ However, the study only compared 3 kinds of traditional CMIs, but there are 13 kinds of CHIs commonly used in clinical treatment for AECOPD, so the study cannot meet the actual clinical needs. In addition, not do the analysis performed the GRADE evaluating to report the certainty of the evidence. As a result, we will update and improve the literature search strategy based on the NMA. A very broad search strategy will be used in this study, which will ensure any trials with indirect comparisons will be included making this study more comprehensive.

**Table 2:**
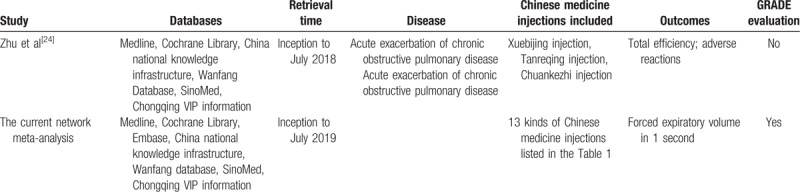
Comparison of basic information between the current and the other network meta-analysis.

### 3.2 Insufficient and prospect

Due to strict inclusion/exclusion criteria defined, potential biases will be minimized. In this study, 3 issues need special attention. First, we found that most studies published in Chinese journals rarely achieve strictly design randomization in our experience. Therefore, most of the randomized controlled trials included in this study will likely be rated as low quality. Secondly, this study is a secondary literature analysis rather than a direct face-to-face comparative study. NMA will be used indirectly using a common comparator to estimate the relative validity between the different CMIs. The inherent problem in meta-analysis is the existence of heterogeneity that affects estimation. It comes from the diversity of clinical, the methodologic features, and the differences between the studies. Thirdly, as a new and emerging statistical method, NMA inevitably has some limitations and imprecisions. Research design, network connectivity, and overall test similarity are 3 conditions for a good NMA. Although inclusion/exclusion criteria have been defined to limit heterogeneity, this may still exist. Therefore, meta-regression will be used to assess the impact of study level covariates to minimize this. Finally, sensitivity analysis will need to be conducted to show the robustness of results as well as the appropriate use of prior distributions when fitting Bayesian random effects GLMs. As this study is the secondary research based on literature, it is not necessary for ethics approval and patient consent. This protocol is designed in accordance with guidelines for NMA protocols.^[25]^ It will be based on the PRISMA extension statement for NMA.^[10]^ The results of the NMA will be submitted to a peer-reviewed journal upon completion.

This study will provide useful information for the evaluation of AECOPD's current effective CMIs. These results will provide clinicians with information, provide optimal CMI, fill evidence gaps, and identify promising CMIs in future trials.

## Author contributions

**Conceptualization:** Wenjiang Zheng, Tianqi Gao.

**Data curation:** Zhihui Zhou, Qian Yan.

**Investigation:** Wenjiang Zheng, Huiting Huang, Yu Hong, Tiange Zhang.

**Methodology:** Wenjiang Zheng, Tianqi Gao, Huili Liao.

**Project administration:** Wenjiang Zheng, Xiaohong Liu.

**Resources:** Wenjiang Zheng, Tiange Zhang.

**Supervision:** Xiaohong Liu.

**Writing – original draft:** Wenjiang Zheng, Tianqi Gao, Zhihui Zhou, Qian Yan, Yu Hong, Tiange Zhang.

**Writing – review & editing:** Wenjiang Zheng, Xiaohong Liu.

Xiaohong Liu orcid: 0000-0003-4795-3039.
